# ProBDNF promotes sepsis-associated encephalopathy in mice by dampening the immune activity of meningeal CD4^+^ T cells

**DOI:** 10.1186/s12974-020-01850-0

**Published:** 2020-05-28

**Authors:** Ru-Yi Luo, Cong Luo, Feng Zhong, Wei-Yun Shen, Hui Li, Zhao-Lan Hu, Ru-Ping Dai

**Affiliations:** 1 Department of Anesthesiology, The Second Xiangya Hospital, Central South University, Central Ren-Min Road No. 139, Changsha, Hunan Province China; 2 Anesthesia Medical Research Center, Central South University, Changsha, China; 3 Department of Anesthesiology, Guangdong Cardiovascular Institute, Guangdong General Hospital, Guangdong Academy of Medical Sciences, Guangzhou, China

**Keywords:** Sepsis, Encephalopathy, Meningeal immunity, CD4^+^ T cells, Brain-derived neurotrophic factor precursor, Monoclonal antibody

## Abstract

**Background:**

Sepsis-associated encephalopathy (SAE) increases the mortality of septic patients, but its mechanism remains unclear. The present study aimed to investigate the roles of T lymphocytes, proBDNF, and their interaction in the pathogenesis of SAE.

**Methods:**

Fear conditioning tests were conducted for cognitive assessment in the lipopolysaccharide (LPS, 5 mg kg^−1^)-induced septic mice. Meninges and peripheral blood were harvested for flow cytometry or qPCR. FTY720 and monoclonal anti-proBDNF antibody (McAb-proB) were used to investigate the effect of lymphocyte depletion and blocking proBDNF on the impaired cognitive functions in the septic mice.

**Results:**

In the septic mice, cognitive function was impaired, the percentage of CD4^+^ T cells were decreased in the meninges (*P* = 0.0021) and circulation (*P* = 0.0222), and pro-inflammatory cytokines were upregulated, but the anti-inflammatory cytokines interleukin (IL)-4 (*P* < 0.0001) and IL-13 (*P* = 0.0350) were downregulated in the meninges. Lymphocyte depletion by intragastrically treated FTY720 (1 mg kg^−1^) for 1 week ameliorated LPS-induced learning deficit. In addition, proBDNF was increased in the meningeal (*P* = 0.0042) and peripheral (*P* = 0.0090) CD4^+^ T cells. Intraperitoneal injection of McAb-proB (100 μg) before LPS treatment significantly alleviated cognitive dysfunction, inhibited the downregulation of meningeal (*P* = 0.0264) and peripheral (*P* = 0.0080) CD4^+^ T cells, and normalized the gene expression of cytokines in the meninges. However, intra-cerebroventricular McAb-proB injection (1 μg) did not have such effect. Finally, exogenous proBDNF downregulated the percentage of CD4^+^ T cells in cultured splenocytes from septic mice (*P* = 0.0021).

**Conclusion:**

Upregulated proBDNF in immune system promoted the pathogenesis of SAE through downregulating the circulating CD4^+^ T cells, limiting its infiltration into the meninges and perturbing the meningeal pro-/anti-inflammatory homeostasis.

## Introduction

Sepsis-associated encephalopathy (SAE) is a diffuse or multifocal cerebral dysfunction caused by systemic inflammation without evidence of direct brain infection. The incidence of SAE is reported to range from 8 to 70% [1, 2]. Encephalopathy increases the mortality rate in the septic patients [3]. The mortality rate of SAE patients has been reported to be from 26 to 49% higher than that of septic patients without neurological manifestations [4]. Several mechanisms have been proposed regarding the pathogenesis of SAE, these include the dysfunction of blood-brain barrier, the effect of endotoxins, the effect of inflammatory mediators [5, 6], neurovascular coupling [7], regulation of vagus nerve [8], and the vicious cycle between brain injury and a progressively aberrant immune response [9]. Nonetheless, there is a lack of effective clinical intervention for SAE currently. It is still urgent to identify the mechanism of the pathogenesis of SAE as the target for treatment.

Recently, accumulating evidence has indicated that the meningeal immune response is involved in learning and memory [10]. The meninges are three membranes, the dura mater, arachnoid mater, and the pia mater. Their primary function is to protect the central nervous system (CNS). The meninges are also reported to be an important part of inflammatory microenvironment related to CNS functions [11]. A Morris water maze training was found to improve cognitive function in normal mice, accompanied by increased infiltration of CD4^+^ T cells and interleukin (IL)-4 expression in the meninges; deletion of IL-4 abrogated the enhanced memory induced by training [10, 12]. Meningeal T cell-derived IL-13 also facilitated learning behavior in mice [12]. Despite the critical role of meningeal CD4^+^ T cells in cognitive function in healthy mice, the functions of meningeal T lymphocytes in the development of SAE remain elusive.

Brain-derived neurotrophic factor precursor (proBDNF) is the precursor protein of mature BDNF. Not only as an intermediate of mature BDNF, proBDNF can act on its high affinity receptor, pan neurotrophin receptor 75 (p75^NTR^) and exert opposite biological functions to mature BDNF in the CNS. For example, unlike the neuroprotective effect of mature BDNF, proBDNF-p75^NTR^ signaling promotes neuronal apoptosis and axonal pruning, and negatively regulates learning and memory [13]. In addition to the nervous system, proBDNF is also expressed in the immune cells. For example, brain neuroinflammation upon parasite infection induced the upregulation of proBDNF signaling in the monocytes/macrophages [14]. Our previous study established that proBDNF and p75^NTR^ were highly upregulated in the nerve fibers and inflammatory cells in footpad injected with formalin in mice. Neutralizing the upregulated endogenous proBDNF by monoclonal blocking antibody (McAb-proB) greatly attenuated pain hypersensitivity after peripheral inflammation [15]. Our more recent study further reported that LPS injection induced the upregulation of proBDNF in the T cells of the mesenteric lymph node [16]. Therefore, it is plausible that proBDNF may modulate functions of immune cells and may be involved in the pathogenesis of SAE.

The present study hypothesized that proBDNF-mediated CD4^+^ T lymphocyte dysfunction in the meninges was implicated in the pathogenesis of SAE. In attempt to test this hypothesis, the present study firstly aimed to investigate the role of meningeal T lymphocytes in the development of SAE in mice with the use of fear conditioning paradigm. Next, the present study further explored the role of proBDNF in the immune system and its effect on the pathogenesis of SAE.

## Materials and methods

### Animals

Male C57BL/6 mice (12 to 14 weeks old) were obtained from Central South University Animal Services (Changsha, China). All experimental mice were housed six per cage with food and water ad libitum. Animals were kept in a 12:12 light:dark cycle, 21 ± 1 °C temperature and 50 ± 10% humidity. The experimental protocol was approved by the Animal Care and Use Committee of Central South University (Approval No. 2018334) and conformed to the National Institutes of Health Guide for the Care and Use of Laboratory Animals. All efforts were made to minimize the number of mice used and their sacrifice.

### Drugs and treatment

LPS derived from *Escherichia coli* serotype 055:B5 ( Sigma-Aldrich, USA, catalog: L2880, 5 mg kg^−1^) was dissolved in 0.9% saline (0.3 ml) and injected intraperitoneally to mice for induction of SAE model [17]. Control animals were injected with equivalent volumes of saline. The animals were randomly divided into control group and LPS group. The time that mice received intraperitoneal injection of LPS as well as killed are between 9 and 11 a.m.

FTY720 (Melonepharma, China, catalog: 162359-56-0) were used for eliminating peripheral blood lymphocytes and therefore reducing meningeal infiltration of lymphocytes as reported before [10]. Briefly, animals were treated daily with an oral administration (1 mg kg^−1^ in 0.1 ml saline by gavage) of FTY720 starting at 1 week before LPS injection for 7 days and followed with treatment throughout the fear conditioning test. The proper time and dosage of FTY720 used was based on a previous study by Kipnis et al. [10]. Mice subjected to intragastric administration of equal saline with same protocol were used for control.

For investigating the role of monoclonal anti-proBDNF antibody (McAb-proB), mice were treated with intraperitoneal injection of 100 μg in 0.3 ml McAb-proB at 30 min before the induction of SAE model, or bilateral intracerebroventricular delivery (i.c.v) of 1 μg in 1 μl McAb-proB 3 days before LPS injection. The biological activity and safety of McAb-proB have been characterized by our previous studies [15, 16, 18, 19]. Mouse IgG (CMCTAG, catalog: AT1596) were used for isotype control of related experiments. The dosage of McAb-proB used for treatment is followed by our previous research [15, 16, 18].

### Fear conditioning test

Fear conditioning test was used to evaluate LPS-induced cognitive dysfunction in mice. Fear conditioning test was performed in a plastic chamber equipped with a stainless-steel grid floor. Mice was stayed in the chamber for 5 min for adaptation and followed by fear conditioning acquiring test 1 day after LPS injection. The paired conditioned and unconditioned stimulus used in the test is as follows: 60 dB white noise for 20 s and 0.45 mA foot shock for 1 s, and the foot shock was given in the last 1 s of the white noise. For fear conditioning acquisition, mice were conducted with the above stimulus for 5 times, by which separated by 40-s intervals. One day after acquiring fear conditioning, mice were placed in the same chamber for 5 min for contextual fear conditioning memory detection without other stimuli. One day after that mice were then put in another chamber with different visual and tactile cues for cued fear conditioning memory detection, but with the same white noise stimuli for four times. The freezing time of mice in the chamber, which is indicated by not having any body movement except breath, was calculated for analysis. The researcher who counted the freezing time of mice by video was blind to the experimental design.

### Meningeal single cell and splenocyte isolation

Single-cell suspension from the meninges of mice were isolated as introduced by Jonathan Kipnis [20]. Specifically, mice were anesthetized with lethal dose of pentobarbital sodium (90 mg kg^−1^) and transcardially perfused with around 20 ml 0.9% saline after fresh blood was collected from ventricle. Head of mice were removed and the skulls were stripped of all flesh. Then, mandibles, skull material rostral to maxillae, and skull tops, cutting clockwise, beginning and ending inferior to the right post-tympanic hook were successively removed. After that, brains and the superior skulls were kept in ice-cold 0.1 M PBS. All the meninges, including the dura, arachnoid, and pia mater materials were then collected from the interior aspect of skulls and surfaces of brains. Meninges from 4 mice were pooled together as one group, which were gently smashed by plastic plungers and passed through 70-μm nylon mesh cell strainers (Life Science, USA, catalog: 156187). The collected meningeal single-cell suspension then was washed once with ice-cold 0.1 M PBS at 400 RCF at 4 °C for 10 min. Cells were resuspend in 100 μl 0.1 M PBS for flow cytometry staining.

Spleen was collected from mice after saline perfusion, then they were gently smashed and pressed through 70-μm nylon mesh cell strainers with 4 ml PBS. In total, 50 μl of the single-cell suspension was used as splenocyte for further flow cytometry staining.

### Flow cytometry

Meningeal single cell, splenocyte, and 100 μl fresh blood were stained for flow cytometry analysis. Cells were stained for extracellular markers with antibody cocktail of zombia aqua fixable viability dye conjugated to brilliant violet 510 (Biolegend, USA, catalog: B279913); CD45 conjugated to APC-CY7 (Biolegend, USA, catalog: 103116); CD3 conjugated to PE-CY7 (Biolegend, USA, catalog: 100220); CD4 conjugated to PER-CP5.5 (Biolegend, USA, catalog: 100540); CD8 conjugated to APC (Invitrogen, USA, catalog: 1953015); CD11b conjugated to brilliant violet421(BD Biosciences, USA, catalog: 562605); and CD19 conjugated to PE (Biolegend, USA, catalog: 100508) for 30 min at 4° C. Then cells were washed by 2 ml 0.1 M PBS at 400 RCF at 4 °C for 10 min for 3 times. For fresh blood and splenocyte, red blood cells were then removed by immersed cells in lysis buffer (Invitrogen, USA, catalog: 2076712) at room temperature for 10 min and followed by three washes as above. After that, cells were read by flow cytometer (Cytotek, USA).

ProBDNF staining was conducted as our previous study [21]. Washed cells that had been stained with above extracellular markers were fixed by fixation buffer (Invitrogen, USA, catalog: 2060489) for 20 min at room temperature and followed by permeabilization as introduced by the manufacturer protocol (Invitrogen, USA, catalog: 2009161). After three washes by 0.1 M PBS at 400 RCF at 4 °C for 10 min, they were stained with humanized monoclonal antibodies to proBDNF (10 ng ml^−1^, obtained from Shanghai Yile Biotechnology Corp., its biological function has been proved before [15]) or human IgG control (10 ng ml^−1^, Abcam, UK, catalog: ab37385) for 30 min at room temperature. Cells were then washed by 1 × permeabilization buffer and stained with sheep anti-human IgG conjugated to FITC (Abcam, UK, catalog: ab6896). After rinse, cells were read by the flow cytometer (Cytotek, USA), and data were analyzed with FlowJo^TM^ 10.4 software.

### Meningeal cell counts

The absolute counting microsphere kit (BD, USA, catalog: 340334) was used for meningeal cell count as introduced before [22]. Pooled single-cell suspensions of meninges from four mice of each group were eventually diluted to 250 μl and stained for the appropriate markers as described above in the counting tubes. The absolute count of the cell population is acquired by dividing the number of positive cell events by the number of bead events, and then multiplying by the bead concentration. The final absolute number of cells in meninges in each mouse is then divided by four. The experiments are repeated for four times for final statistical analysis.

### Intracerebroventricular (i.c.v.) delivery

Mice were anesthetized with an intraperitoneal injection of pentobarbital sodium (40 mg kg^−1^) and placed in a stereotaxic apparatus (RWD, Shenzhen, China) with bregma and lambda at the horizontal level. The procedure was followed as Sarah L. DeVos described [23]. Specifically, stereo positioning coordinates of bregma: anteroposterior − 0.1 mm, mediolateral ± 0.8 mm, dorsoventral − 2.2 mm, were used for bilateral ventricular injection in mice (2nd edition, Keith B.J. Franklin and George Paxinos) [24]. Humanized monoclonal anti-proBDNF antibody or isotype IgG (1 μg μl^−1^, 0.5 μl volume in each side, over 10 min) were dissolved in sterile saline and injected to mice bilaterally. Three days later, all mice behaved normally and were conducted with i.p. injection of 5 mg kg^−1^ LPS.

### RNA isolation and RT-PCR

Meninges were collected as described before. Total RNA of the meninges isolated from four mice in the same group was extracted by TRIzol reagent (QIAGEN Science, USA, catalog: 79306). RNA (0.2 μg) was then quantified by a clinx software (Shanghai, China) and reversely transcribed into cDNA by Reverted Aid First Strand cDNA Synthesis Kit (Thermo Fisher Scientific, USA, catalog: 00727497). Quantitative real-time PCR was performed with SYBR Green (Bio Rad, USA, catalog: TCS0803) on CFX96 Touch™ Deep Well Real-Time PCR Detection system (Bio-Rad Laboratories, Inc., USA). The primer sequences were showed below:

**Table:** 

Gene	Primer	Sequence
GAPDH	Forward	5′-TGCCTCGCTTCACCACCTTCT-3′
	Reverse	5′-AGGCCGGTGCTGAGTATGTC-3′
IL-1β	Forward	5′-TGGATGCTCTCATCAGGACAG-3′
	Reverse	5′-GAAATGCCACCTTTTGACAGTG-3′
IL-6	Forward	5′-AGTTGTGCAATGGCAATTCTGA-3′
	Reverse	5′-CTCTGGCTTTGTCTTTCTTGTTATCTTT-3′
IL-4	Forward	5′-GGTCTCAACCCCCAGCTAGA-3′
	Reverse	5′-GCCGATGATCTCTCTCAAGATAT-3′
IFN-γ	Forward	5′-GCTTTGCAGCTCTTCCTCAT-3′
	Reverse	5′-TTTGCCAGTTCCTCCAGATA-3′
CD4	Forward	5′-TCCTAGCTGTCACTCAAGGGA-3′
	Reverse	5′-TCAGAGAACTTCCAGGTGAAGA-3′
IL-13	Forward	5′-CAGGTGTGCTCCATTTCATTCTAAT-3′
	Reverse	5′-ATGGCTTTTGTGCATATCAGATGCT-3′

Relative mRNA levels were calculated using the 2^−ΔΔCT^ method and normalized to *GAPDH* in the same sample. The ct number of *GAPDH* between groups in our experiments is stable and around 16~18.

### Immunofluorescence

Dissection and immunostaining of whole-mount meninges of mice were performed as described by Antoion [25]. The whole meninges were mounted and washed for 3 times with 0.1 M PBS. After blocking by 5% BSA and 0.2% triton at 37 °C for 30 min, slices were stained with humanized monoclonal anti-proBDNF antibody (2 ng ml^−1^) overnight at 4 °C as introduced by our previous study [19]. Then, they were washed by 0.1 M PBS for 3 times and incubated with goat anti-human secondary antibody (1:400, USA, catalog: SA00003-12) for 2 h at 37 °C. After wash, immunofluorescence mounting buffer containing DAPI (Vector Laboratories, USA, catalog: ZE0815) was used for covering. A confocal microscope (NIKON Eclipse Ti, Tokyo, Japan) was used for imaging.

### Cell culture

Splenocytes were resuspended in DMEM supplemented with 10% heat-inactivated FBS, 2 mM glutamine, 25 mM HEPES (all from Gibco, USA), 5 × 10^−5^ M 2-ME (Solarbio, China), and 100 U ml^−1^ penicillin. 4 × 10^5^ cells were put in each well of a 96-well flat-bottom plate and stimulated with 100, 200, or 500 ng ml^−1^ proBDNF protein (Alomone Labs, Israel, catalog: B243) as introduced by our previous studies [21], respectively. 72 h later, splenocytes were collected and analyzed by flow cytometry as indicated above.

### Statistical analysis

All data are presented as mean ± SEM. An unpaired two-tailed Student’s *t* test was applied to compare two independent groups. One-way ANOVA followed by Tukey post hoc test was used to analyze multiple groups. Repeated measures ANOVA or two-way ANOVA was used to analyze repeated measurements and followed by Bonferroni post hoc test. Statistical values were defined as significant at *P* < 0.05. The diagrams and statistical analysis were conducted by using GraphPad 7 software (GraphPad Software, USA).

## Results

### Alterations of meningeal immune activity in LPS-induced cognitive dysfunction in mice

**Fig. 1:**
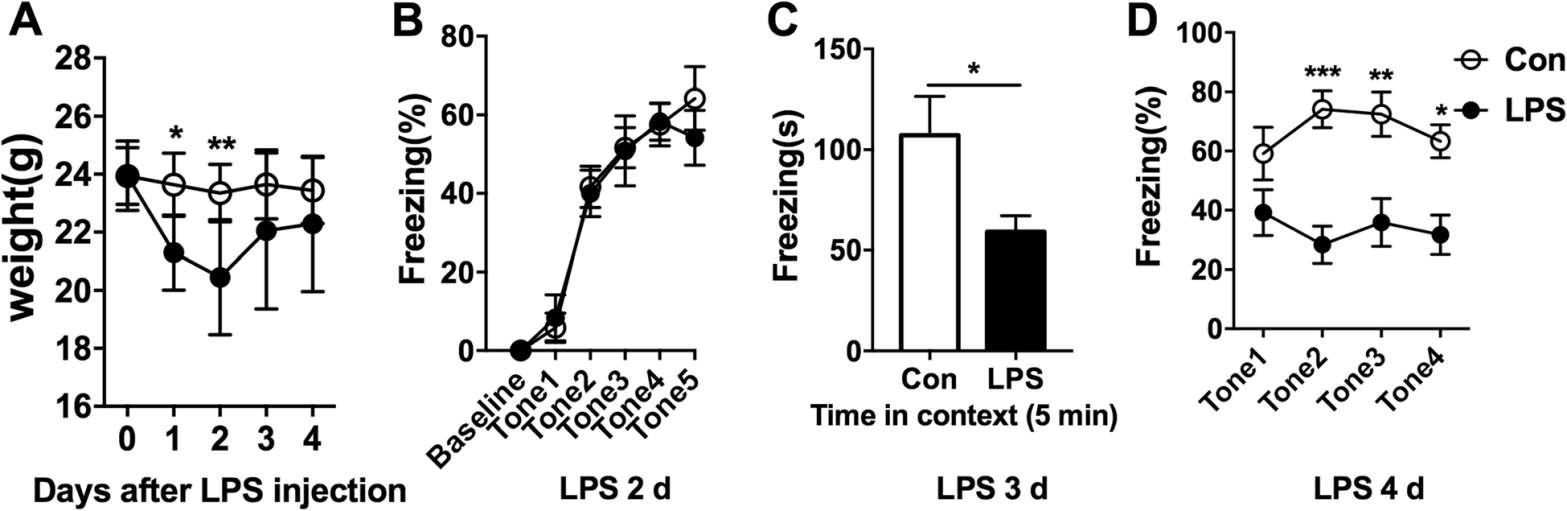
Impaired cognitive dysfunction after lipopolysaccharide (LPS) injection. Mice were i.p. injected with LPS (5 mg kg^−1^) and behavior tests were conducted at 1 day after LPS injection. **a** There was a reduction in body weight between saline-treated and LPS-injected mice. *n* = 8 each group. The data was analyzed by repeated measures ANOVA followed by Bonferroni post hoc test, *P < 0.05, **P < 0.01. **b** LPS injection did not affect acquisition of fear conditioning test in mice. *n* = 8 each group. **c***,***d** Decreased freezing time in (**c**) contextual and (**d**) cued fear conditioning test in LPS-treated mice indicated that 5 mg kg^−1^ LPS peritoneal injection induced cognitive dysfunction in mice. *n* = 8 each group. Data **c** was analyzed by unpaired T test and data **b** and **d** were analyzed by repeated measures ANOVA followed by Bonferroni post hoc test, *P < 0.05, **P < 0.01, ***P < 0.001. Data are presented as mean ± SEM. Con = saline injected control. LPS = i.p. LPS injection. i.p. = intraperitoneal

We first established the sepsis mouse model with i.p. injection of 5 mg kg^−1^ LPS. With this dose, a reduction of weight was detected in mice 1 and 2 days after LPS injection, but not in the following observing days [Fig. 1a, F(1,13) = 26.26; *P =* 0.0002]. Next, we examined the fear conditioning memory after LPS injection. As shown in Fig. 1 b[F(1,10) = 0.093;*P* = 0.766], there was no significant difference in the acquisition of fear conditioning memory between the septic mice and the controls. However, the freezing time in the context of fear conditioning memory was far shorter in septic mice than in control mice [Fig. 1c, *t*(10) = 2.287; *P* = 0.045]. In the cued fear conditioning memory, the rate of freezing in different trials was also much lower in the septic mice than in controls [Fig. 1d, F(1.10) = 18.77; *P =* 0.0015]. These findings confirmed that sepsis impairs the maintenance of fear conditioning memory, as previously reported [26].

**Fig. 2:**
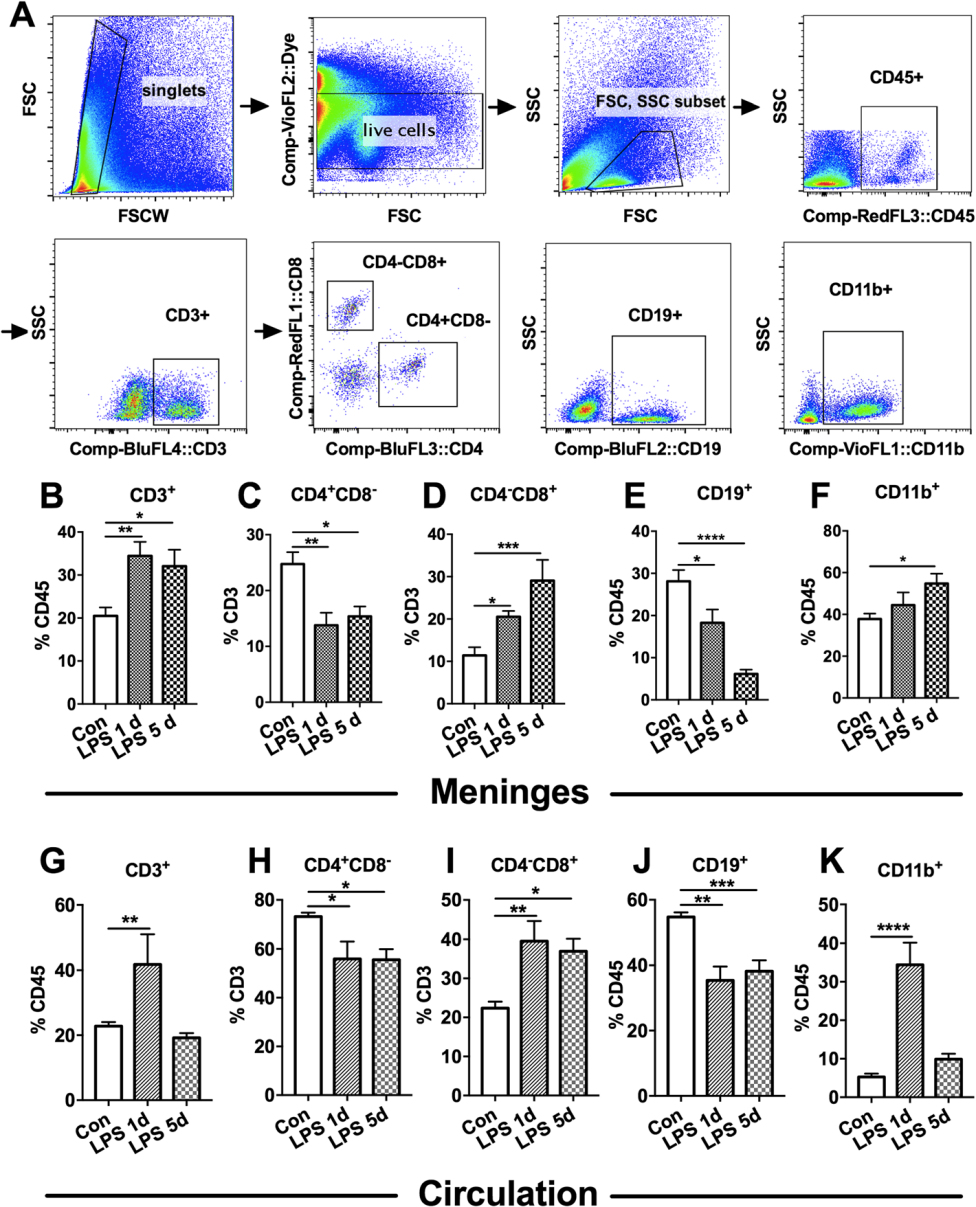
Alterations in meningeal and peripheral immune cells in the septic mice. Mice were i.p. injected with LPS (5 mg kg^−1^) and were sacrificed for the measurement of meningeal and peripheral immune cells by flow cytometry analysis. **a** Representative gating scheme of flow cytometry for meningeal immune cells. **b** Meningeal CD3^+^ T cells increased at 1 day and 5 days in LPS-treated mice relative to saline control. **c**, **d** LPS injection in mice decreased the percentage of (**c**) CD4^+^ T cells but increased the percentage of (**d**) CD8^+^ T cells in CD3^+^ T cells in the meninges. **e** Percentage of CD19^+^ B cells in CD45^+^ cells in the meninges was downregulated but (*F*) CD11b^+^ cells in the meninges was increased in mice given LPS injection as compared to saline injected control. *n* = 8 in the Con group and *n* = 6 in the LPS group. **g**–**k** LPS induced significantly increased percentage of **g** CD3^+^ T cells, **i** CD8^+^ T cells, and **k** CD11b^+^ cells among CD45^+^ cells, but decreased percentage of **h** CD4^+^ T cells and **j** CD19^+^ B cells in peripheral blood. *n* = 5 in each group. Data were analyzed by one-way ANOVA and followed by Tukey post hoc test, *P < 0.05, **P < 0.01, ***P < 0.001, ****P < 0.0001. Data are presented as mean ± SEM. Con = saline injected control. LPS 1 d = 1 day after i.p. LPS injection. LPS 5 d = 5 days after i.p. LPS injection. i.p. = intraperitoneal

Immune cells in the meninges were examined after LPS injection by flow cytometry (Fig. 2a). As shown, the percentage of CD3^+^ T cells among CD45^+^ cells were higher in the meninges at 1 day and 5 days after LPS injection than in the saline-injected control mice [Fig. 2b, F(2,27) = 7.722; *P* = 0.0022]. Among T lymphocytes, the subpopulations of CD4^−^CD8^+^ cytotoxic T (Tc) cells in meninges were increased (Fig. 2d), but the percentage of CD4^+^CD8^−^ T helper (Th) cells in the meninges were significantly decreased (Fig. 2c) at 1 day [14.9% ± 6.0% of CD3, mean ± SD, F(2,27) = 8.423; *P* = 0.0021] and 5 days (15.4% ± 4.6% of CD3, *P* = 0.0140) after LPS injection when compared to the saline injected control mice (27.8% ± 5.0% of CD3). After LPS injection, the percentage of CD19^+^ B cells among CD45^+^ cells was decreased [Fig. 2e, F(2,25) = 13.25; *P* = 0.0001] while the percentage of CD11b^+^ monocytes/macrophages increased [Fig. 2f, F(2,27) = 3.358; *P* = 0.0498] in the meninges. These findings suggested that LPS injection induced diverse pattern of meningeal immune cells infiltration.

Similar to changes in the meninges, CD3^+^ T cells, CD4^−^CD8^+^ Tc cells and CD11b^+^ monocytes/macrophages were increased, while CD4^+^CD8^−^ Th cells [73.3% ± 3.0% of saline control, 55.9% ± 2.3% of LPS 1 d and 55.6% ± 7.5% of LPS 5 d, F(2,27) = 7.722; *P* = 0.0022] and CD19^+^ B cells were decreased in peripheral blood in mice after LPS injection (Fig. 2g–k).

### Distinct cytokine genes expression in the meninges after LPS injection

**Fig. 3:**
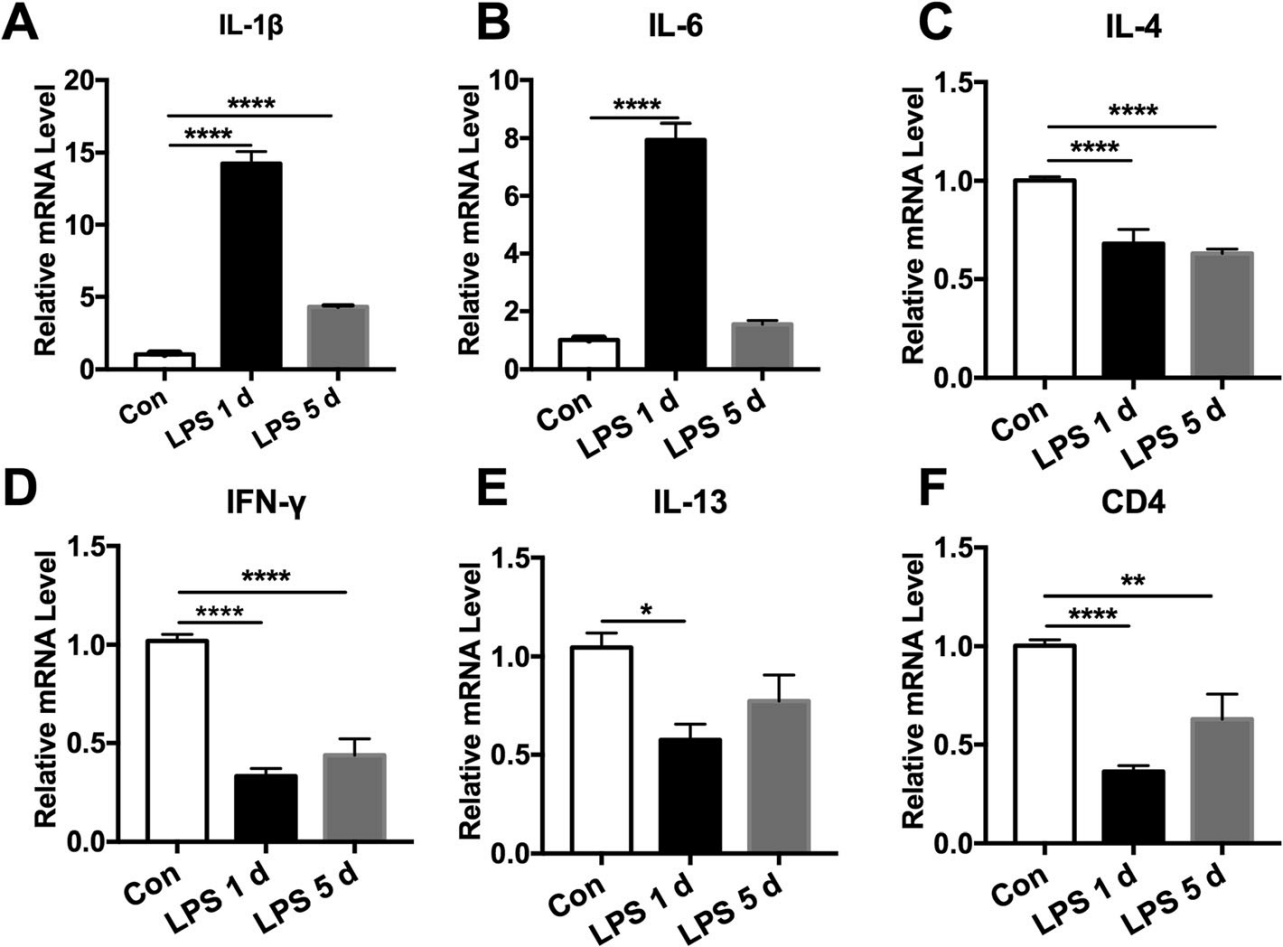
Distinct gene expression of cytokines in meninges after lipopolysaccharide (LPS) injection. Mice were i.p. injected with LPS (5 mg kg^−1^), and meninges were collected for qPCR. The meninges of control mice were harvested at 24 h after they were injected with saline. **a**, **b** Upregulation of IL-1β and IL-6mRNA levels in the meninges after LPS injection. **c**–**e** Meningeal **c** IL-4, **d** IFN-γ, and **e** IL-13 mRNA levels were greatly downregulated after LPS injection. **f** The gene level of CD4 in the meninges was significantly lower at 1 day and 5 days of LPS injected mice than in saline control. *n* = 6 in each group. Experiments were performed in triplicate. Data were analyzed by one-way ANOVA and followed by Tukey post hoc test, *P < 0.05, **P < 0.01, ****P < 0.0001. Data are presented as mean ± SEM. Con = saline injected control. LPS 1 d = 1 day after i.p. LPS injection. LPS 5 d = 5 days after i.p. LPS injection. i.p. = intraperitoneal

Next, we examined the expression pattern of inflammatory cytokines in the meninges after LPS injection in mice. As shown, the expression of meningeal IL-1β and IL-6 gene was dramatically increased at 1 day after LPS injection [Fig. 3a, F(2,15) = 1362; *P* < 0.0001 and b, F(2,9) = 638.2; *P* < 0.0001], of which IL-1β remained high as of 5 days in LPS-treated mice relative to saline control (Fig. 3a). Given the role of CD4^+^CD8^−^ Th cells on cognitive dysfunction, we then also analyzed IL-4, interferon (IFN)-γ and IL-13 gene alterations in the meninges. Remarkably, the gene expression of meningeal IL-4, IFN-γ, and IL-13 were all dramatically lower than in controls at 1 day (1.5 ± 0.3, 3.4 ± 0.9, 1.9 ± 0.6 fold) and 5 days (1.5 ± 0.1, 2.6 ± 0.9, 1.5 ± 0.4 fold) after LPS injection [Fig. 3c, F(2,15) = 57.62; *P* < 0.0001; d, F(2,21) = 92.63; *P* < 0.0001; e, F(2,6) = 5.706, *P* = 0.041]. Consistently, the expression of CD4 gene was also downregulated at 1 day [2.8 ± 0.6 fold, F(2,15) = 14.83; *P* = 0.005] and 5 days [2.0 ± 0.9 fold, F(2,15) = 14.83;*P* = 0.006] after LPS injection (Fig. 3f). These findings suggest that sepsis upregulated the pro-inflammatory cytokines but specifically downregulated the CD4^+^CD8^−^ Th cells-related cytokines in the meninges.

### Depletion of meningeal T lymphocytes attenuated the impaired fear memory in the septic mice

**Fig. 4:**
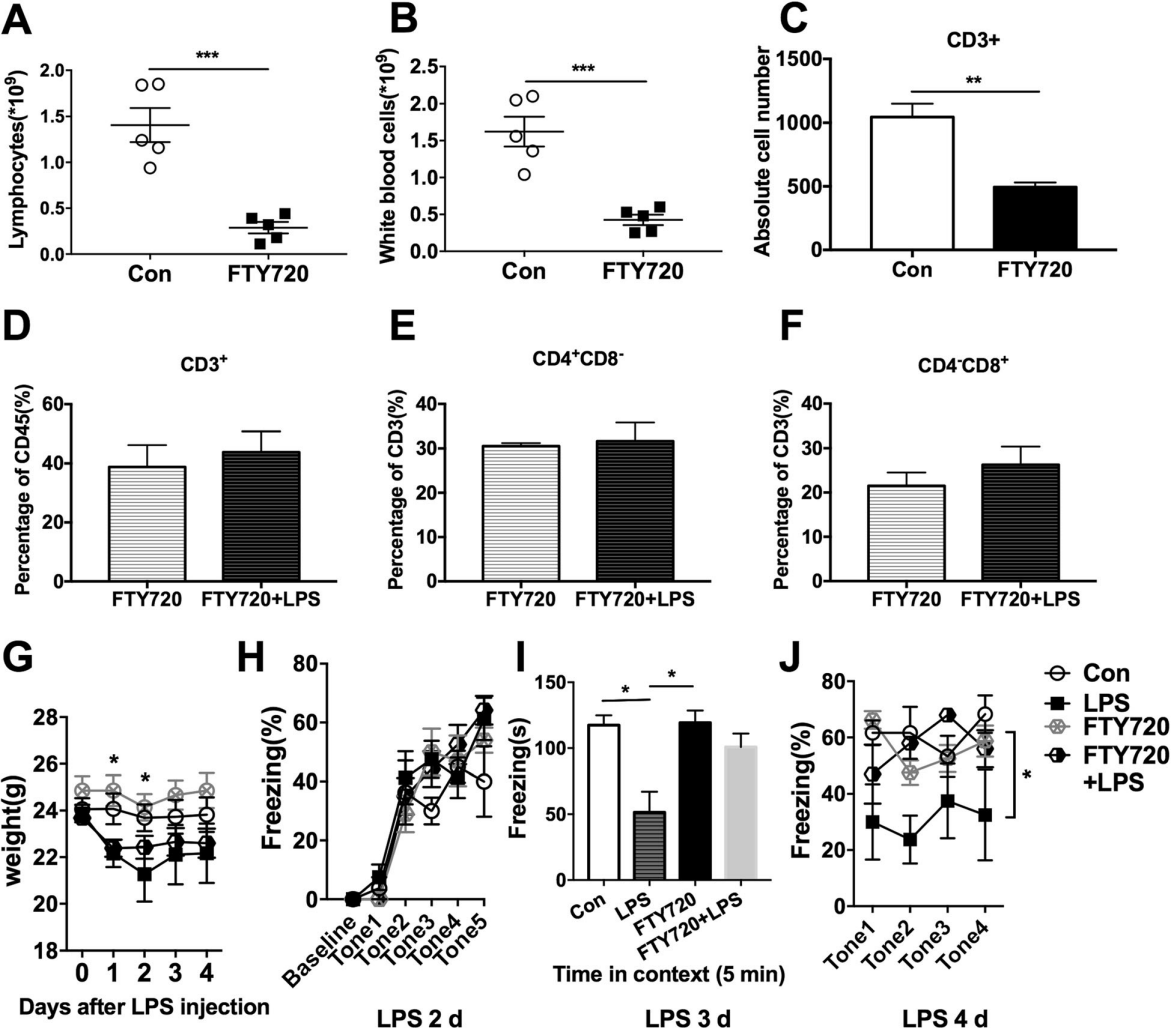
FTY720 pretreatment alleviated lipopolysaccharide (LPS)-induced fear conditioning memory deficit. Mice were intragastrically treated with FTY720 daily for a week followed by i.p. LPS (5 mg kg^−1^) injection. Behavior tests were conducted and peripheral blood and meninges were collected at 1 day after LPS injection. **a**, **b** FTY720 significantly cleared **a** lymphocytes and **b** white blood cells in peripheral blood. *n* = 5 in each group. **c** The absolute number of CD3^+^ T cells in the meninges in FTY720-treated mice were dramatically lower than in saline-treated controls. **d***–***f** Neither the percentage of **d** CD3^+^ T cells in CD45^+^ cells nor the percentage of **e** CD4^+^ T cell or **f** CD8^+^ T cells in CD3^+^ T cells changed after LPS injection in FTY720 pre-treated mice. *n* = 5 in each group. Data **a**–**f** were analyzed by unpaired *T* test, **P < 0.01, ***P < 0.001. **g** FTY720 alone did not influence weight changes induced by LPS injection in mice. **h** FTY720 treatment did not influenced the fear conditioning acquiring, but it greatly reversed the reduced freezing time in the **i** contextual and **j** cued fear conditioning test induced by LPS injection. *n* = 6 in each group. Data **g**, **h**, and **j** were analyzed by two-way ANOVA and followed by Tukey post hoc test and data **i** was analyzed by one-way ANOVA and followed by Tukey post hoc test, *P < 0.05. Data are presented as mean ± SEM. Con = saline control. i.p. = intraperitoneal

To establish the role of meningeal lymphocytes in the impaired cognitive functions of the septic mice, we used FTY720 to clear peripheral lymphocytes and therefore decreased the recruitment of meningeal lymphocytes. FTY720 hampers the entry of lymphocytes into efferent lymphatics within lymph nodes, thereby delaying their subsequent return to circulation [27]. Administration of FTY720 apparently cleared the peripheral lymphocytes [1.4 ± 0.4 vs 0.3 ± 0.1 × 10^9^, *t*(8) = 5.703; *P* = 0.0005] (Fig. 4a). Notably, administration of FTY720 also almost cleared white blood cells because lymphocytes make up the majority of the white blood cells in mice [Fig. 4b, *t*(8) = 5.57; *P* = 0.0005]. As shown in Fig. 4c, FTY720 treatment significantly reduced the number of CD3^+^ T cells in the meninges [1045.7 ± 104.4 vs 494.0 ± 36.5, F(3,9) = 37.41; *P* = 0.0010]. FTY720 treatment abrogated LPS-induced elevated levels of CD3^+^ T cells in CD45^+^ cells in the meninges (Fig. 4d). Furthermore, after FTY720 pretreatment, LPS injection did not alter the percentage of meningeal CD4^+^CD8^−^ Th cells [Fig. 4e, F(3,12) = 3.421; *P* = 0.701] or CD4^−^CD8^+^ Tc cells in CD3^+^ T cells [Fig. 4f, F(3,12) = 5.384; *P* = 0.948]. This is probably due to the reduction of total T lymphocytes in the meninges. These results indicate that FTY720 treatment reversed the alterations in meningeal immune cell infiltration induced by LPS.

Next, we determined whether depletion of lymphocytes affects cognitive function after LPS injection. FTY720 pretreatment neither affect weight in healthy nor in LPS-treated mice by itself [Fig. 4g, F(3,24) = 14.46, *P* < 0.0001 ], it also did not affect the acquisition of fear conditioning training [Fig. 4h, F(3,15) = 1.412, *P* = 0.278]. In the normal mice, administration of FTY720 was not associated with any difference in contextual fear conditioning memory relative to saline treatment (Fig. 4i). However, FTY720 pretreatment significantly ameliorated sepsis-induced impaired contextual fear conditioning memory as indicated by the restored freezing time [Fig. 4i, F(3,13) = 6.452, *P* = 0.0065]. Similarly, FTY720 treatment alone did not affect cued fear conditioning memory but rendered the decrease in relative freezing time less pronounced in septic mice [Fig. 4j, F(3,12) = 3.661, *P* = 0.044]. These results strongly suggest that depletion of peripheral lymphocytes by FTY720, which subsequently reduced the meningeal T lymphocytes infiltration into the meninges, attenuated the impaired cognitive function induced by sepsis.

### Upregulation of proBDNF in the meningeal and peripheral immune cells in the septic mice

**Fig. 5:**
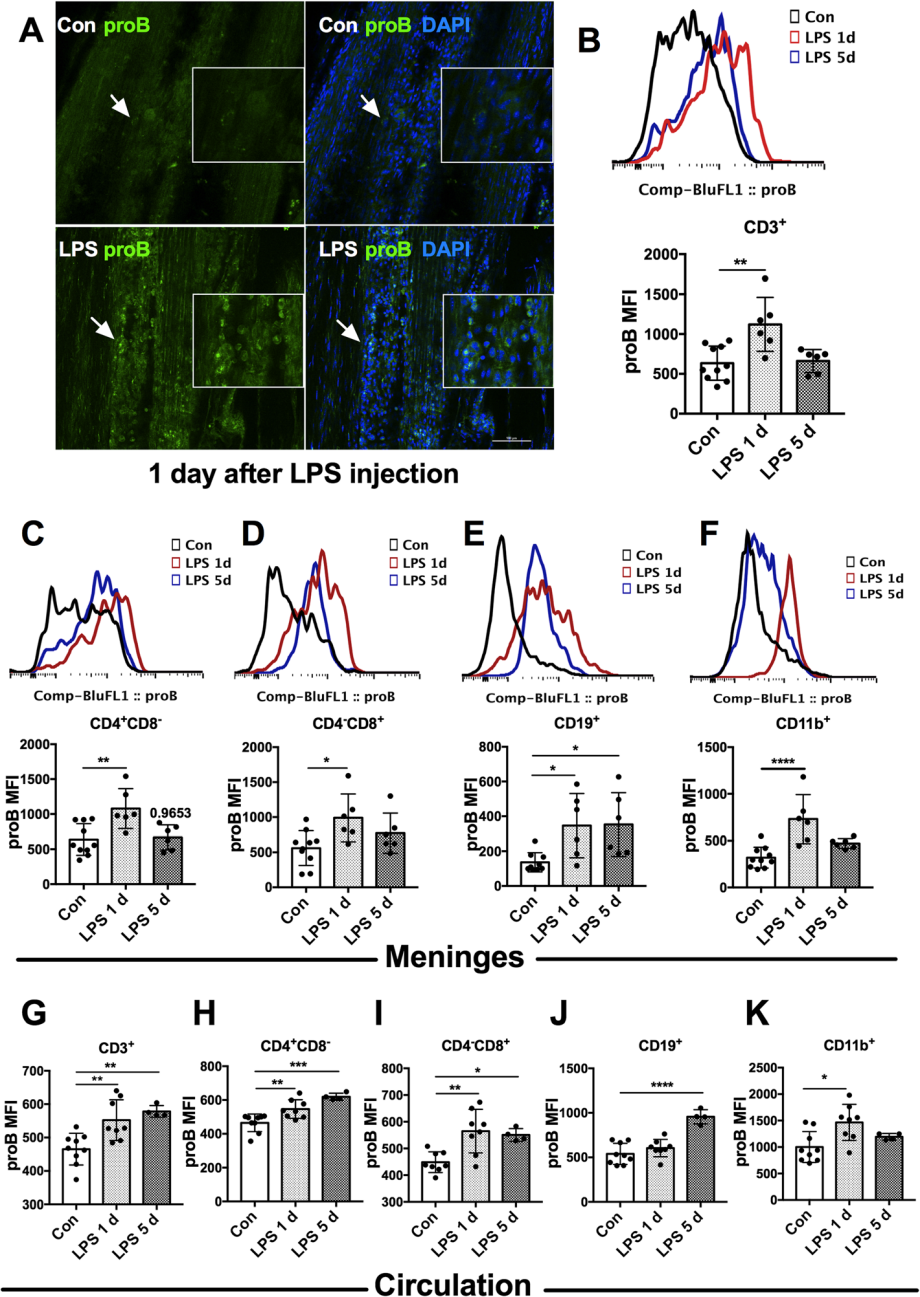
Increased proBDNF expression in meningeal and peripheral blood immune cells in septic mice. Mice were i.p. injected with LPS (5 mg kg^−1^) and meninges and peripheral blood were harvested for immunofluorescence staining or flow cytometry. **a** Representative whole mount meningeal immunofluorescence images showed markedly increased proBDNF-positive staining cells in the meninges in mice at 1 day after LPS injection compared to saline injected mice. The high magnification images around the arrows are displayed in a white square as insets. Bar = 100 μm. **b***–***f** Representative meningeal single cell flow cytometry images (*upper panel*) and its statistical analysis (*lower panel*) indicated that proBDNF MFI was increased in meningeal **b** CD3^+^ T cells, **c** CD4^+^ T cells, **d** CD8^+^ T cells, and **f** CD11b^+^monocytes/macrophages at 1 day after LPS injection. proBDNF in meningeal **e** CD19^+^ B cells upregulated until 5 days after LPS injection. *n* = 10 in the Con group, *n* = 6 in LPS groups. **g***–***k** Upregulation of proBDNF in **g** CD3^+^ T cells, **h** CD4^+^ T cells, **i** CD8^+^ T cells, **j** CD19^+^ B cells, and **k** CD11b^+^ monocytes/macrophages in peripheral blood in LPS-injected mice were detected. *n* = 9 in Con group, *n* = 8 in the LPS1d group, *n* = 4 in the LPS 5d group. Data **b**–**k** were analyzed by one-way ANOVA and followed by Tukey post hoc test, *P < 0.05, **P < 0.01, ***P < 0.001, ****P < 0.0001. Data are presented as mean ± SEM. proB = proBDNF, Con = saline injected control. LPS 1d = 1 day after i.p. LPS injection. LPS 5d = 5 days after i.p. LPS injection. i.p. = intraperitoneal. MFI = mean fluorescence intensity

Whole mount immunofluorescence of the meninges was performed to assess the expression of meningeal proBDNF in the sepsis. As shown in Fig. 5a, sparse proBDNF-positive staining was detected in the control mice. However, the expression of proBDNF was significantly increased in meningeal sagittal sinus at 1 day after LPS injection. Next, we used flow cytometry to examine the cellular localization of proBDNF in the septic mice. As shown in Fig. 5b–f, proBDNF mean fluorescence intensity (MFI) was upregulated in all tested subtypes of immune cells in the meninges, including CD3^+^ T cells [1.6 ± 0.2 fold, F(2,19) = 8.623;*P* = 0.002], CD4^+^CD8^−^ Th cells [1.7 ± 0.3 fold, F(2,19) = 7.572;*P* = 0.0038], CD4^−^CD8^+^ Tc cells [2.0 ± 1.0 fold, F(2,19) = 4.355; *P* = 0.0277], CD19^+^ B cells [3.0 ± 1.6 fold, F(2,18) = 5.827;*P* = 0.0112] and CD11b^+^ monocytes/macrophages [2.0 ± 0.5 fold, F(2,19) = 12.82; *P* = 0.0003] 1 day after LPS injection (Fig. 5b–f). At 5 days after LPS treatment, the MFI of proBDNF was comparable to the saline-injected control in CD3^+^ T cells, CD4^+^CD8^−^ Th cells, CD4^−^CD8^+^ Tc cells (Fig. 5b–d), and CD11b^+^ monocytes/macrophages (Fig. 5f). However, proBDNF MFI in the CD19^+^ B cells was still higher than that of saline control (Fig. 5e).

The upregulation of meningeal proBDNF in septic mice prompted us to assess the expression of proBDNF in peripheral blood cells. Flow cytometry analysis showed that proBDNF expression was persistently increased in CD3^+^ T cells, CD4^+^CD8^−^ Th cells, and CD4^−^CD8^+^ Tc cells at 1 day [all showed 1.2 ± 0.2 fold, F(2,18) = 9.766; *P* = 0.0058, F(2,18) = 14.51; *P* = 0.0090 and F(2,18) = 8.704; *P* = 0.0028] and 5 days (all showed 1.2 ± 0.1 fold, F(2,18) = 9.766, *P* = 0.0039; F(2,18) = 14.51, *P* = 0.0002 and F(2,18) = 8.704; *P* = 0.0296) after LPS injection (Fig. 5g–i). The increase in proBDNF in CD19^+^ B cells appeared only at 5 days [1.6 ± 0.3 fold, F(2,18) = 22.66; *P* < 0.0001] in the septic mice (Fig. 5j), and peripheral CD11b^+^ monocytes/macrophages showed the increase of proBDNF at 1 day [1.5 ± 0.4 fold, F(2,18) = 5.463;*P* = 0.0105] after LPS injection and they returned to base level thereafter (Fig. 5k). These results strongly suggest a pathogenic role for proBDNF in the immune systems of mice in LPS-induced cognitive dysfunction.

### Systemic McAb-proB treatment alleviated the impaired fear memory and meningeal pro-inflammatory microenvironment in septic mice

**Fig. 6:**
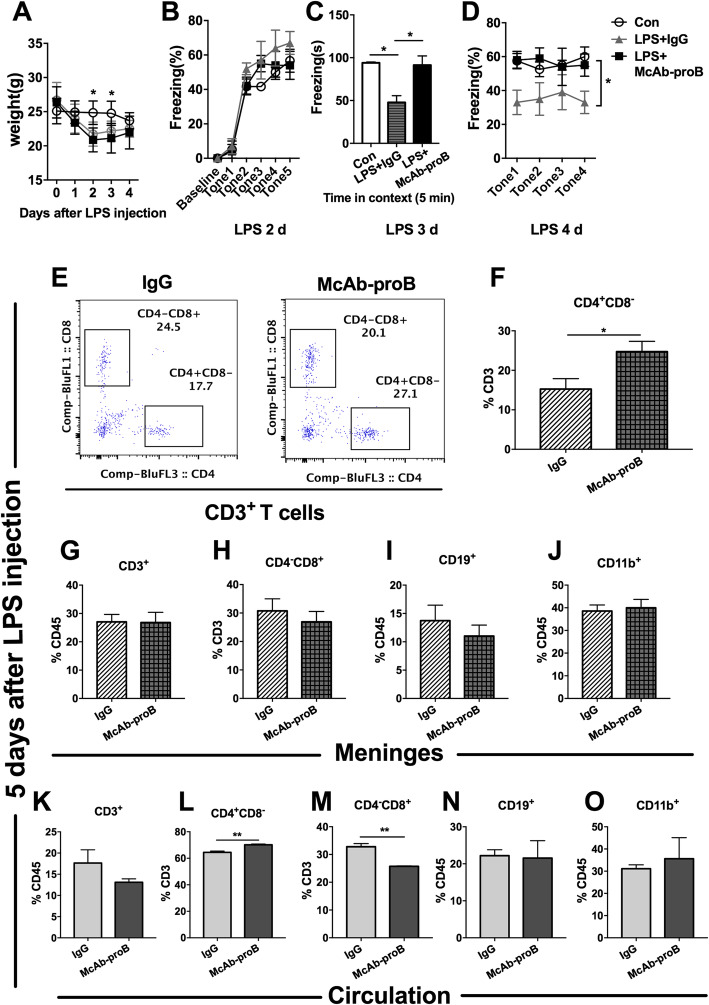
Systemic blockade of proBDNF ameliorated cognitive dysfunction and restored meningeal and peripheral CD4^+^ T cell ratio in septic mice. Mice were i.p. injected with proBDNF 30 min before LPS (5 mg kg^−1^) injection. Fear conditioning testing was performed 1 day after LPS injection. Meninges and peripheral blood were harvested 5 days after LPS injection for flow cytometry. **a** McAb-proB did not influence the weight of mice or **b** fear conditioning acquiring. **c***,***d** McAb-proB greatly alleviated memory deficit induced by LPS injection in mice as indicated by the increased freezing time in (**c**) contextual and (**d**) cued fear conditioning tests in the McAb-proB group relative to the IgG control. *n* = 8 in each group. Data **a**, **b**, and **d** were analyzed by repeated measures ANOVA and followed by Bonferroni post hoc tests. Data **c** was analyzed by one-way ANOVA and followed by Tukey post hoc test, *P < 0.05. **e***–***f** Representative meningeal **e** single cell flow cytometry and **f** analysis of its results indicated that McAb-proB greatly increased the percentage of CD4^+^ T cells among CD3^+^ T cells than IgG control in LPS injected mice. **g***–j* McAb-proB did not change the infiltration of immune cells such as **g** CD3^+^ T cells, **h** CD8^+^ T cells, **i** CD19^+^ B cells, and **j** CD11b^+^monocytes/macrophages in the meninges after LPS injection relative to IgG control. *n* = 4 in each group. **k***–***o** McAb-proB greatly reversed the decreased percentage of **l** CD4^+^ T cells and the increased percentage of **m** CD8^+^ T cells in CD3^+^ T cells, but had no effect on percentage of **k** CD3^+^ T cells, **n** CD19^+^ B cells, or **o** CD11b^+^ monocytes/macrophages in CD45^+^ cells in the peripheral blood of septic mice. *n* = 6 in each group. Data **f**–**o** were analyzed by unpaired *T* test, *P < 0.05, **P < 0.01, ****P < 0.0001. Data are presented as mean ± SEM. Con = saline injected control, McAb-proB = monoclonal anti-proBDNF antibody injection, IgG = isotype Igg injected control

To verify the role of proBDNF in the immune system on memory regulation, we i.p. injected monoclonal anti-proBDNF antibody (McAb-proB) into mice before LPS treatment. The dose of 100 μg McAb-proB was found to fully block the biological function of the endogenous proBDNF without apparently affecting the behaviors in naïve mice [15, 16, 18, 19]. Systemic administration of McAb-proB did not affect the weight changes induced by LPS injection (Fig. 6a) or acquisition of fear conditioning memory [Fig. 6b, F(2,10) = 2.783; *P* = 0.109]. However, McAb-proB treatment did greatly ameliorate the impaired context and cued fear conditioning memory in the septic mice [Fig. 6c, F(2,9) = 7.424;*P* = 0.0125, and d, F(2,11) = 4.297; *P* = 0.0418]. To further investigate the association of improved memory with the infiltration of immune cells in the meninges and peripheral blood, flow cytometry was performed to examine the percentage of different populations of immune cells. McAb-proB treatment significantly restored the percentage of CD4^+^CD8^−^ Th cells in CD3^+^ T cells in the meninges to the normal level, but IgG treatment did not [24.5% ± 8.2% vs 15.2% ± 6.9%, *t*(15) = 2.462; *P* = 0.0264] (Fig. 6e–f). However, it did not change the infiltration of CD3^+^ T cells, CD4^−^CD8^+^ Tc cells, CD19^+^ B cells, or CD11b^+^ monocytes/macrophages in the meninges after LPS injection (Fig. 6g–j). Similarly, McAb-proB dramatically elevated the percentage of CD4^+^CD8^−^ Th cells [70.2% ± 1.0% vs 64.4% ± 1.8%, *t*(4) = 4.913; *P* = 0.0080] but reduced CD4^−^CD8^+^ Tc cells [25.7% ± 0.3% vs 32.8% ± 2.0%, *t*(4) = 6.172; *P* = 0.0035] in peripheral blood in LPS-treated mice (Fig. 6k–o).

**Fig. 7:**
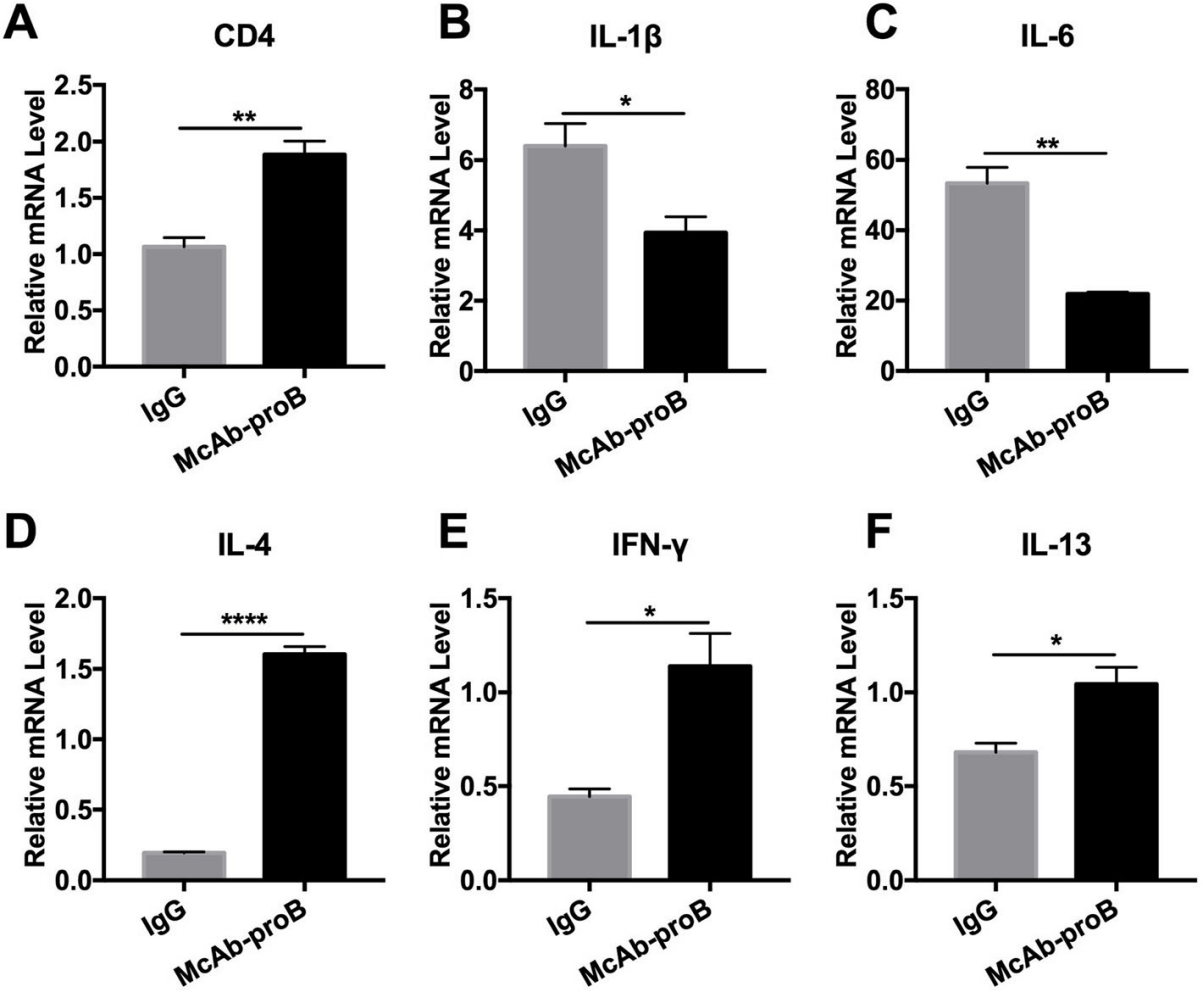
Systemic blockade of proBDNF restored meningeal pro-inflammatory microenvironment in septic mice. Mice were i.p. injected with proBDNF 30 min before LPS (5 mg kg^−1^) injection. Meninges were harvested 5 days after LPS injection for qPCR. **a** The level of CD4 gene expression was higher in the meninges of the McAb-proB group than in IgG controls in septic mice. **b–f** Gene levels were significantly lower in **b** IL-1β and **c** IL-6 but higher in **d** IL-4, **e** IFN-γ, and **f** IL-13 in the meninges after LPS injection in the McAb-proB group as compared to IgG control. *n* = 5 in each group. All experiments were performed at least in triplicate. Data were analyzed by unpaired *T* test, *P < 0.05, **P < 0.01, ****P < 0.0001. Data are presented as mean ± SEM. McAb-proB = monoclonal anti-proBDNF antibody injection, IgG = isotype Igg injected control

qPCR showed that systemic McAb-proB treatment reversed the downregulation of CD4 mRNA expression in the meninges 5 days after LPS injection [Fig. 7a, *t*(4) = 5.644, *P* = 0.0049]. The activation of IL-1β and IL-6 genes was significantly inhibited by McAb-proB treatment relative to IgG treatment [Fig. 7b, *t*(4) = 3.192, *P* = 0.033 and c, *t*(4) = 6.938, *P* = 0.0023]. The downregulation of the IL-4, IFN-γ, and IL-13 genes in septic mice was also dramatically reversed in the meninges by McAb-proB pre-treatment [Fig. 7d, *t*(4) = 25.15, *P* < 0.001, e, *t*(4) = 3.892; *P* = 0.177, and f*, t*(4) = 3.574; *P* = 0.023]. These results indicated that blocking the upregulated proBDNF restored the decrease of CD4^+^CD8^−^ Th cell infiltration and inhibited pro-inflammatory microenvironment in the meninges induced by LPS injection.

### Effect of i.c.v. McAb-proB injection on the impaired fear memory and meningeal pro-inflammatory microenvironment in septic mice

**Fig. 8:**
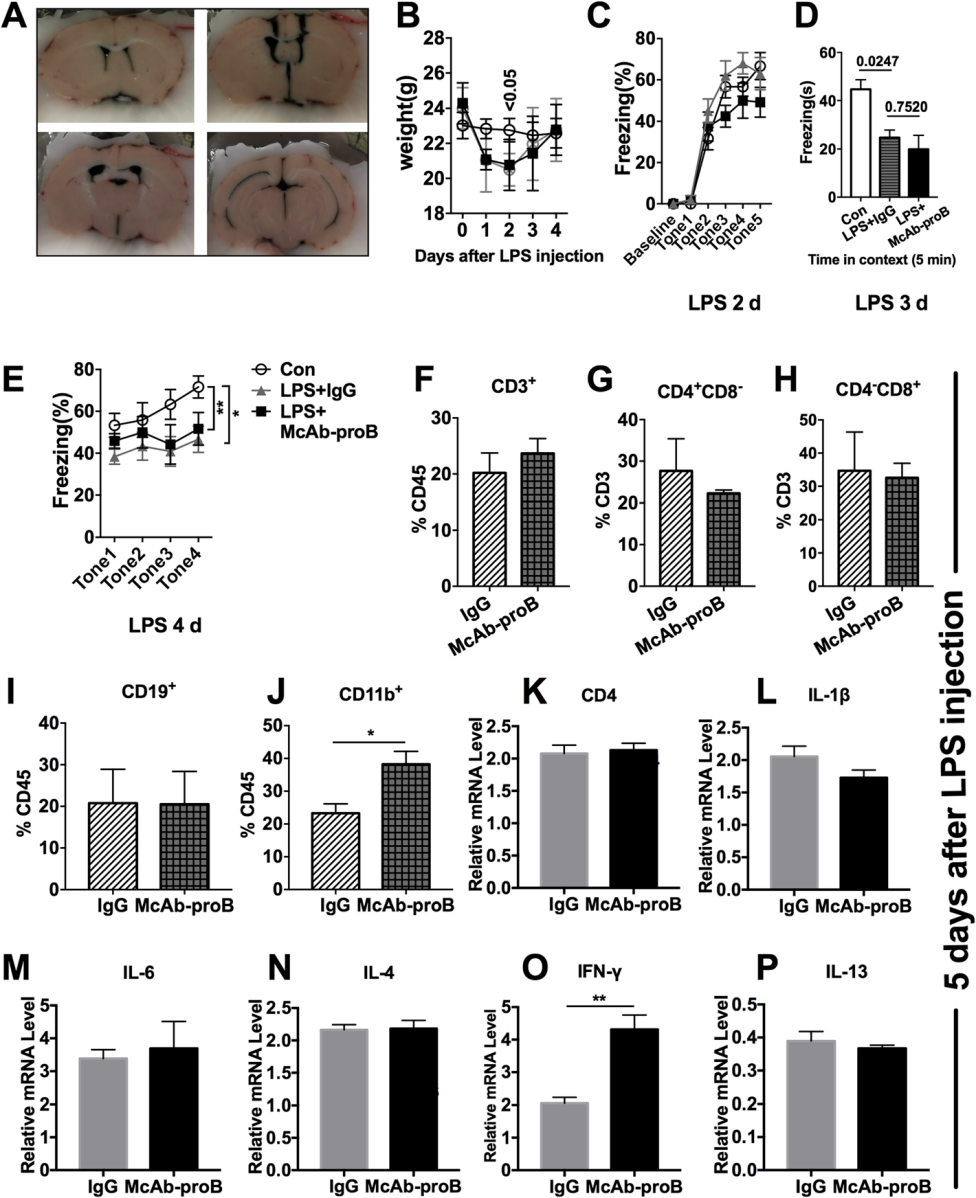
Effect of i.c.v. injection of anti-proBDNF antibody on fear memory and meningeal immune activity in the septic mice. Mice were bilateral i.c.v. injected with 1 μg McAb-proB 3 days before LPS injection. Behavior tests were performed 1 day after LPS injection. **a** Representative images showed the broad and thorough diffusion of drugs in cerebroventricular lumen following i.c.v. injection with methylene blue. **b** McAb-proB i.c.v. injection did not influence weight of mice. **c–e** There was no difference of **c** fear conditioning acquiring performance in each group, nor McAb-proB increased the freezing time of **d** contextual or **e** cued fear conditioning test as compared to IgG control after LPS injection. *n* = 6 in each group. Data **b**, **c**, and **e** were analyzed by repeated measures ANOVA and followed by Bonferroni post hoc test and data **d** was analyzed by one-way ANOVA and followed by Tukey post hoc test, *P < 0.05, **P < 0.01. **f–p** Meninges were harvested 5 days after LPS injection for flow cytometry analysis and qPCR. **f–j** Flow cytometry indicated that McAb-proB i.c.v. injection did not alter immune cell subpopulation, except for the greater percentage of **j** CD11b^+^ monocytes/macrophages in CD45^+^ cells in the meninges, as compared to IgG control. *n* = 4 in each group. **k–p** No difference in CD4 gene level or gene expression of IL-1β, IL-6, IL-4, IFN-γ, and IL-13 in the meninges between McAb-proB-injected mice and isotype-injected control after LPS injection. *n* = 5 in each group. Experiments were performed at least in triplicate. Data **f**–**p** were analyzed by unpaired *T* test, *P < 0.05, **P < 0.01. Data are presented as mean ± SEM. Con = saline injected control, McAb-proB = monoclonal anti-proBDNF antibody injection, IgG = isotype Igg injected control, i.c.v. = intracerebroventricular

The therapeutic effect of McAb-proB on impaired cognitive function may be due to direct action on the meningeal immune cells. It has been reported that i.c.v. antibody can spread to the third ventricle of mice, which causes antibody to become distributed throughout the microvasculature of the cerebral and cerebellar hemispheres [22]. McAb-proB 1 μg per mice was i.c.v. injected before LPS treatment and behavioral studies was performed thereafter (Fig. 8a). As shown in Fig. 8b–e, there was no difference in context or cued conditioning memory between the IgG and McAb-proB-treated groups, indicating that blocking meningeal proBDNF alone did not improve the impaired cognitive function. In addition, i.c.v. McAb-proB treatment had little effect on alterations in meningeal immune cells induced by LPS injection (Fig. 8f–j) except for an increased percentage of CD11b^+^ monocytes/macrophages Fig. 8j, *t*(2) = 9.607; *P* = 0.0119]. Furthermore, there was not a significant effect on the CD4 or cytokine gene expression after i.c.v. McAb-proB treatment (Fig. 8k–p), except for increased expression of meningeal IFN-γ [Fig. 8o, *t*(4) = 4.76; *P* = 0.009]. These results indicated that systemic proBDNF played a critical role in the development of LPS-induced cognitive dysfunction.

### Exogenous proBDNF decreased CD4^+^CD8^−^ Th cells but increased CD4^−^CD8^+^ Tc cells in the cultured splenocytes

**Fig. 9:**
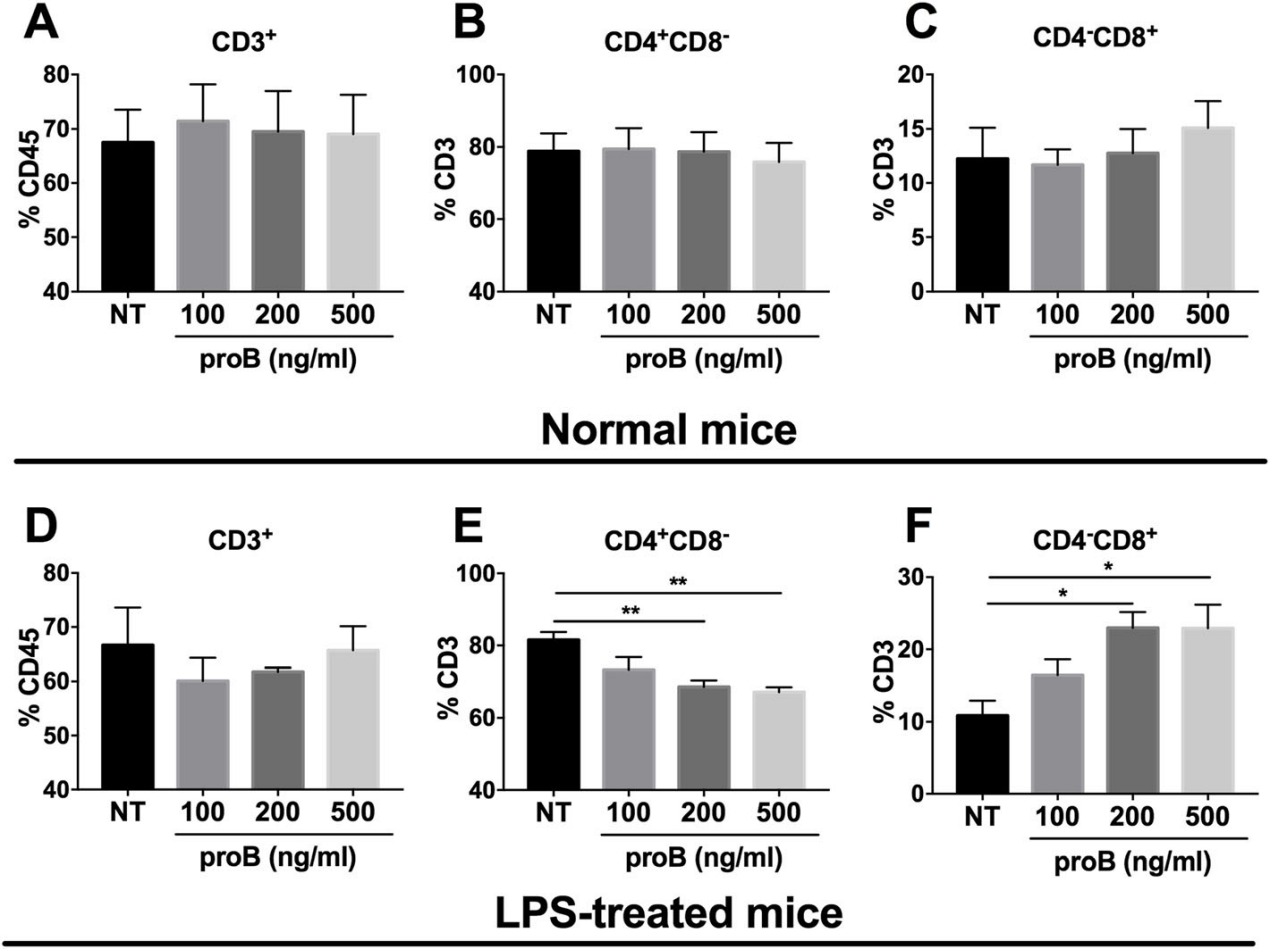
Exogenous proBDNF protein reduced CD4^+^ T cells but increased CD8^+^ T cells of septic mice in vitro. Mice injected with saline or LPS (5 mg kg^−1^) for 5 days and the splenocytes were isolated and cultured for 3 days in vitro. Exogenous proBDNF did not alter the percentage of **a** CD3^+^ T cells in CD45^+^ cells or the percentage of **b** CD4^+^ T cells or **c** CD8^+^ T cells in CD3^+^ T cells in splenocytes from mice treated with saline. **d** Exogenous proBDNF did not alter the percentage of CD3^+^ T cells in CD45^+^ cells in splenocytes in septic mice. **e–f** ProBDNF treatment significantly decreased the percentage of **e** CD4^+^ T cells but increased the percentage of **f** CD8^+^ T cells in CD3^+^ T cells in splenocytes in LPS-treated mice. *n* = 4 in each group. Data were analyzed by one-way ANOVA and followed by Tukey post hoc test, *P < 0.05, **P < 0.01. Data are presented as mean ± SEM. NT = no treated control. proB = exogenous proBDNF protein treatment

**Fig. 10:**
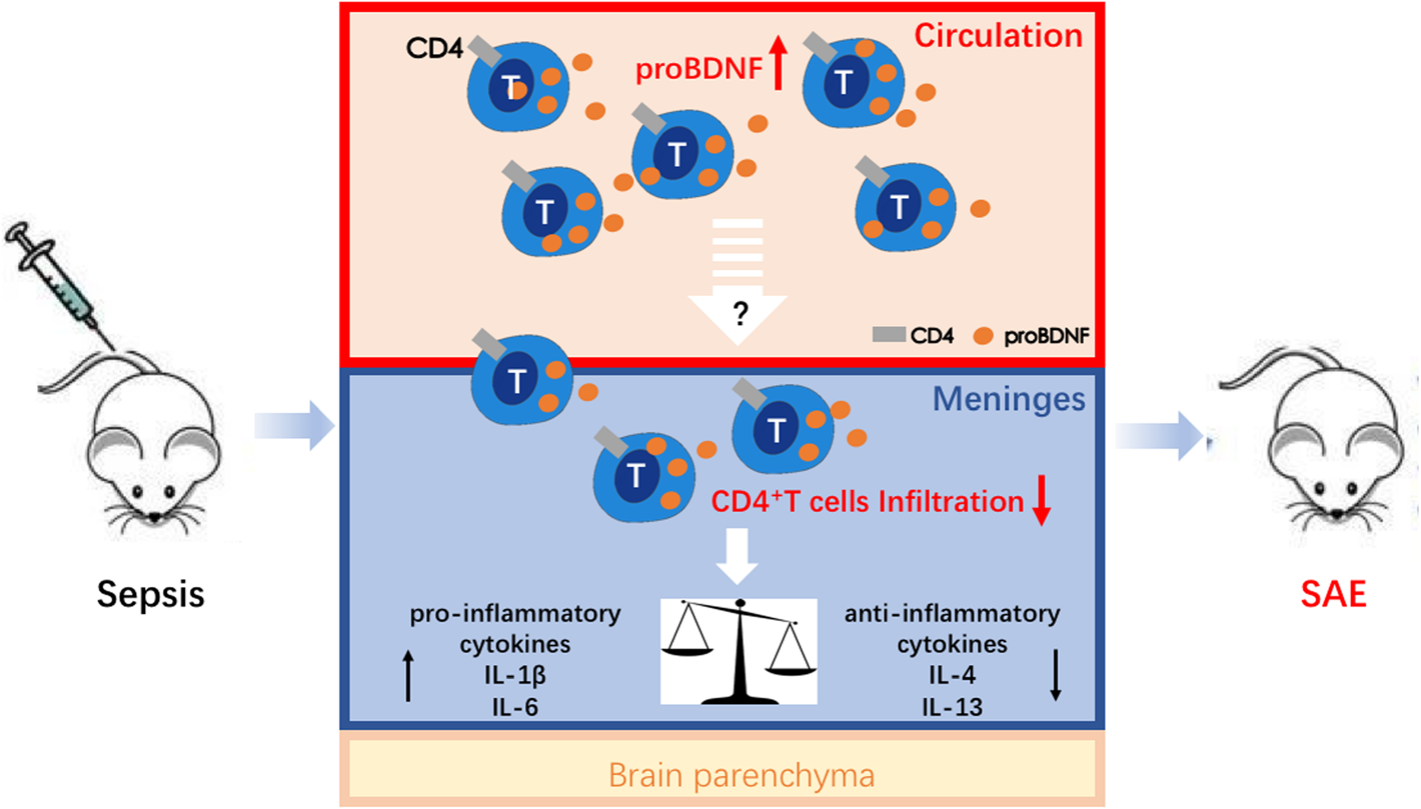
Schematic diagram showing how proBDNF dampens CD4^+^ T cell activity and contributes to the pathogenesis of SAE. In sepsis, proBDNF expression is increased in peripheral blood and meningeal immune cells, which then decreases the infiltration of CD4^+^ T cells in the meninges. As a result, meningeal pro-inflammatory cytokines such as IL-6 and IL-1β are upregulated, but anti-inflammatory cytokines including IL-4 and IL-13 are downregulated, finally leading to SAE. SAE, sepsis-associated encephalopathy

To establish the key role of proBDNF on alterations of T cell subpopulation, we then harvested splenocytes from normal mice and septic mice at 5 days after LPS injection and treated the cultured splenocytes with exogenous proBDNF protein [23]. As shown, proBDNF protein did not affect T cell subgroups in normal mice (Fig. 9a–c). However, it significantly reduced the percentage of CD4^+^CD8^−^ Th cells [1.2 ± 0.1 fold at 500 ng ml^−1^, F(3,12) = 9.588; *P* = 0.0021] and increased the percentage of CD4^−^CD8^+^ Tc cells [2.2 ± 0.3 fold at 500 ng ml^−1^, F(3,10) = 5.402; *P* = 0.0242] in the splenocytes harvested from the septic mice, in a dose-dependent manner (Fig. 9d–f). These results are consistent with the findings that blocking peripheral proBDNF restored the ratio of meningeal CD4^+^CD8^−^ Th cells, reversed the imbalance in meningeal inflammatory cytokines, and ameliorated cognitive dysfunction after LPS injection (Fig. 10).

## Discussion

Clinically, patients with SAE exhibit acutely altered mental status and usually have higher mortality and morbidity than similar patients without SAE [28]. Several SAE animal models have been established, such as cecal ligation and puncture (CLP) and LPS i.p. injection [29, 30]. Although CLP method has been considered the classical animal model for sepsis [31], LPS-induced endotoxemia is also frequently used to mimic sepsis as it leads to sudden and severe systemic inflammation in rodents [32]. Because it is a bacterial endotoxin, LPS injection could trigger broad and violent immune response through acting on Toll like receptor 4 (TLR4). Many studies have shown that peripheral LPS administration triggers both memory consolidation and reconsolidation deficits in fear conditioning memory in mice [33]. For example, Satomoto et al. reported that LPS (5 mg kg^−1^) was sufficient to induce cognitive dysfunction tested by fear conditioning testing, and without septic shock or multi-organ dysfunction [26]. In the present study, we chose 5 mg kg^−1^ LPS to establish the SAE mouse model. Consistent with previous studies [29, 30], 5 mg kg^−1^ LPS injection severely impaired fear memory as indicated by decreased freezing time in both context and cued fear conditioning testing with reversible changes in weight of the mice. In this case, it is a reliable SAE model for the evaluation of changed cognitive function.

Monocytes/macrophages are the main carriers of TLR4, which recognizes LPS. In the present study, LPS injection induced the activation of CD11b^+^ monocyte/macrophages in the peripheral blood and meninges, accompanied by the upregulation of meningeal pro-inflammatory cytokines. These findings suggest that LPS would render a robust pro-inflammatory response. In addition, there was also a different scenario of CD3^+^ cells and CD11b^+^ cell between the meninges and circulation at 5 days after LPS injection in mice. This is likely due to the changed number of CD45^+^ cells in the circulation. Notably, other types of leukocytes such as neutrophils are also CD45^+^ cells. Neutrophils are increased in the circulation at the later phase of LPS injection and may not infiltrate into the meninges [34, 35]. Therefore, the percentage of CD11b^+^ and CD3^+^ in CD45^+^ cells in the meninges can be different from that in circulation. LPS injection has also been reported to reduce the number of CD4^+^ T cells in the peripheral blood in mice [36]. Our results further showed that LPS injection decreased the percentage of CD4^+^ T cells in the peripheral blood and meninges as well. Concomitantly, the gene expression of CD4 and CD4^+^ T cell-related cytokines, including IL-4, IFN-γ, and IL-13, was also downregulated in the meninges of septic mice. On the contrary, IL-6 and IL-1β mRNA levels were elevated in the meninges in LPS-induced septic mice. The different expressions are probably due to the fact that the meningeal IL-4 and IL-13 are mainly released from CD4^+^ T cells whereas IL-6 and IL-1β can be released from CD11b^+^ cells, neutrophils, and T cells [10, 12]. However, we did not examine the cytokine levels in the meninges at 2, 3, and 4 days after LPS injection in mice. There may be some additional dynamics of inflammatory microenvironment in the meninges in septic mice requiring to study in the future. As reported previously, CD4^+^ T cells are required for strengthening cognitive behavior in normal mice, and mice lacking CD4^+^ T cells lost part of their ability to learn [37]. Therefore, the reduced meningeal CD4^+^ T cells and related cytokine gene expression in our results strongly indicate that CD4^+^ T cells are involved in the SAE induced by LPS injection.

To further explore the role of CD4^+^ T cells in impaired fear memory, we here investigated the effect of the lymphocyte depletion by FTY720 on the impaired cognition induced by LPS injection. After depletion of circulating lymphocytes, the increased infiltration of meningeal CD3^+^ T cells induced by LPS injection was totally inhibited, suggesting a close correlation between circulating lymphocytes and the infiltrated meningeal lymphocytes upon LPS challenge. However, the percentage of meningeal CD4^+^ T cells in CD3^+^ T cells remained unchanged in septic mice when mice were pretreated with FTY720. This is likely due to the remarkable reduction of total CD3^+^T cells in the meninges in LPS-induced septic mice. As a result, FTY720 treatment ameliorated the impaired fear memory after LPS injection. These data, together with those from previous studies, strongly suggest that the meningeal CD4^+^ T cells are responsible for the impaired fear memory in septic mice. Previous studies pointed out that in normal mice, depletion of CD4^+^ T cells in the meninges affects the learning behavior by converting meningeal CD11b^+^ monocytes/macrophages to a pro-inflammatory form [10]. In the present study, the relationship among the reduced CD4^+^ T cells, the increased CD11b^+^ monocytes/macrophages, and pro-inflammatory microenvironments in the meninges in septic mice remains unclear and merits further study. Notably, the number of B lymphocytes was reduced in the peripheral and meninges after LPS injection. The role of reduced CD19^+^ B cells in the SAE is not clear yet and warrants further study in the future.

Another important finding of our study is that the upregulation of proBDNF in the immune cells plays an important role in the pathogenesis of SAE. proBDNF, the precursor of BDNF protein, was initially considered as an intermediate for BDNF synthesis [18]. Later studies showed that proBDNF exerted a function opposite that of BDNF in such CNS diseases as depression and Alzheimer’s. ProBDNF was detected in the immune system as well. Exercise and alcohol can increase proBDNF levels in human peripheral blood mononuclear cells [37]. Our previous studies also demonstrated that peripheral proBDNF might be an inflammatory mediator to regulate inflammatory pain [15, 16]. In the present study, LPS treatment induced the upregulation of proBDNF in the circulating and meningeal immune cells. The increased proBDNF was expressed in all the tested circulating and meningeal immune cells, including T cells, B cells, and monocytes/macrophages. It is worthwhile to know that the expression pattern of proBDNF in T cells are different in the meninges and circulation at day 5 post-LPS injection, when the proBDNF MFI in the meninges returned to the basal level whereas it was still high in the circulation. It is still unknown about the discrepancy. One possibility is that the inflammation is gradually resolved in the meninges. Supporting this assumption, the gene expression of IL-6 and IL-1β in the meninges was also decreased at 5 days after LPS injection. In contrast, our recent study demonstrated that proBDNF in the T cells persistently upregulated in the mesenteric lymph node [16]. In our results, i.p. injection of monoclonal antibody against proBDNF greatly attenuated the impairment in fear memory in the septic mice. Systemic McAb-proB treatment reversed the decrease in the percentage of CD4^+^ T lymphocytes in the meninges and attenuated the impaired fear memory. All of these findings suggest that the therapeutic effect of McAb-proB on impaired fear memory takes place via the meningeal CD4^+^ T cell infiltration. Consistent with this assumption, previous findings have reported that the infiltrated meningeal CD4^+^ T cells contribute to learning and memory in normal mice [10, 12].

The effect of systemic McAb-proB treatment on the percentage of meningeal CD4^+^ T cells and their immune activity may be due to the treatment’s direct effect on meningeal CD4^+^ T cells or to its secondary effect on the circulating T cells. In the present study, i.c.v. administration of McAb-proB did not attenuate the impairment in fear memory or the pro-inflammatory microenvironment induced by LPS injection, suggesting the secondary effect of proBDNF on the meningeal CD4^+^ T cells. In this case, the upregulated proBDNF decreased the peripheral CD4^+^ T cells and subsequently reduced their infiltration in the meninges, which also lost its regulative role on meningeal microenvironment (Fig. 10). In the cultured splenocytes from the septic mice, exogenous proBDNF greatly downregulated the CD4^+^ T cell population, but increased the percentage of CD8^+^ T cells. However, given that the cultured splenocytes are fragile and hard to survive for a long time, the present study only examined the effect of proBDNF on T lymphocytes at 3 days after culture which may really reflect the modulation of proBDNF on CD4^+^ T cell population in vivo. Despite the limitation, these findings still strongly suggest that the upregulated proBDNF promotes the development of SAE through reducing the peripheral CD4^+^ T cells and its infiltration into the meninges. In the present study, we found that i.c.v. injection with McAb-proB increased the percentage of CD11b^+^ cells in the meninges, while i.p. injection of McAb-proB had not such an effect in the circulation. Given that a previous study reported that proBDNF inhibited migration of macrophages into an injured spinal cord [38], it may suggest that central blockade of proBDNF may facilitate CD11b^+^ cells migrating to the meninges.

The present study measured the expression of IL-4, IFN-γ, and IL-13 genes to assess the functions of the meningeal CD4^+^ T cells in septic mice because these cytokines are specifically released from CD4^+^ T cells. IFN-γ in the meninges has been shown to have a protective effect on cognition [39]. IL-4 and IL-13 always exert anti-inflammatory effects on immune activity, and their expression in the meninges is believed to promote learning and memory [10]. After LPS injection, IL-4, IFN-γ, and IL-13 were downregulated in the meninges and reversed by systemic McAb-proB treatment. These findings strongly suggest that the upregulated proBDNF mediates downregulation of meningeal anti-inflammatory cytokines and contributes to LPS-induced SAE. McAb-proB treatment also greatly inhibited gene expression of the pro-inflammatory cytokines IL-1β and IL-6 in the meninges. Taken together, our findings strongly indicate that systemic McAb-proB treatment may normalize the balance of the meningeal pro- and anti-inflammatory microenvironment to maintain cognitive function in the septic mice. In this scenario, during the progress of SAE, the upregulated proBDNF in the immune system inhibits the infiltration of CD4^+^ T cells into the meninges, which subsequently inhibited the release of IL-4, IFN-γ, and IL-13, but promotes the release of IL-1β and IL-6 in the meninges. The imbalanced pro- and anti-inflammatory meningeal microenvironments finally contribute to the progress of SAE (Fig. 10). Further investigations should be performed to establish the mechanism by which proBDNF perturbed pro- and anti-inflammatory balance in the meninges by disrupting the functions of CD4^+^ T cell.

## Conclusion

In summary, the present study showed that sepsis upregulated proBDNF in the immune system, which decreased the meningeal CD4^+^ T cell infiltration and disrupted the balance of meningeal pro- and anti-inflammatory microenvironments and initiated cognitive dysfunction. Targeting proBDNF signaling and CD4^+^ T cells could be a potential therapeutic intervention for SAE.

## Data Availability

All data generated or analyzed during this study are included in this published article (and its supplementary information files).

## Abbreviations

LPS: Lipopolysaccharide
CNS: Central nervous system
ProBDNF: Brain-derived neurotrophic factor precursor
SAE: Sepsis-associated encephalopathy
McAb-proB: Monoclonal anti-proBDNF antibody
CLP: Cecal ligation and puncture
IL: Interleukin
